# Cognitive Decline After Cranial Irradiation: Hoping for a Systematic Application of the Reliable Change Index

**DOI:** 10.3389/fpsyg.2020.573919

**Published:** 2020-10-06

**Authors:** Giorgio Gronchi, Silvia Scoccianti, Andrea Peru

**Affiliations:** ^1^Department of Neuroscience, Psychology, Drug Research and Child's Health - Section of Psychology, University of Firenze, Firenze, Italy; ^2^Radiation Oncology Unit, Azienda Ospedaliero-Universitaria Careggi, University of Firenze, Firenze, Italy

**Keywords:** whole-brain radiotherapy, brain tumors, neurocognitive decline, measurement, reliability, reliable change

## Introduction

Assessing change over time is one of the major uses of neuropsychological practice (Heaton et al., 2001); test–retest procedures, for example, are needed to detect deterioration due to brain alteration or improvements induced by rehabilitation treatments. Among the various statistical approaches, the Reliable Change Index (RCI) is one of the simplest and most appropriate measures for evaluating a change over time of an individual score from both a statistical and a clinical point of view (Zahra and Hedge, 2010). The RCI can be defined as a psychometric criterion aimed at evaluating a potential change over time of an individual score (Zahra and Hedge, 2010). Although the RCI is fairly widespread in neuropsychological literature (Chelune et al., 1993; Sawrie et al., 1996; Maassen, 2004; Hensel et al., 2007), in some subfields, this index is still employed in an unsystematic way. Here we will focus on the cognitive consequences of cranial irradiation for oncological management (Gondi et al., 2010, 2012, 2014; Kim et al., 2018; Acharya et al., 2019; Tringale et al., 2019). Converging literature indicates that the link between cranial irradiation and an increased risk of a posttreatment neurocognitive decline occurring months or years after radiation therapy can be taken for granted. Notwithstanding that technological advances allow to minimize the risk of normal tissue injury, a certain degree of structural and/or functional brain alteration seems to be an inescapable consequence of the radiation treatment of brain tumors (Pazzaglia et al., 2020). Since primary brain tumors usually affect middle-aged people, who are often still working and very active in social life, such an effect has great relevance from clinical and social perspectives. That said, the definition of clear criteria for the administration, scoring, and data analysis of the neuropsychological tests used to detect and evaluate in each patient the presence and the degree of radiation-induced cognitive impairment still remains an open issue. In this opinion paper, after some brief remarks about relatively common methodological problems in this literature, we offer some methodological recommendations, focusing on a wider use of RCI.

## Neuropsychological Assessment

While neuropsychological assessment is widely acknowledged as a highly valid and reliable tool to evaluate nearly all cognitive disorders in the course of normal aging, neurodegenerative diseases, as well as traumatic or vascular injury, the lack of standardized procedures made the diagnosis and the description of the supposed cognitive impairment following cranial irradiation controversial. First of all, various studies enrolled patients with diseases of different clinical stages; while some studies investigated patients who underwent cranial irradiation after surgical excision of primary brain tumors (e.g., Haldbo-Classen et al., 2019), other studies recruited patients with cerebral metastases who also received systematic treatment (e.g., Chang et al., 2009; Brown et al., 2016). Second, the timing of neuropsychological assessment varied widely between studies, with a follow-up extending from a few weeks (e.g., Welzel et al., 2008) to several years (e.g., Klein et al., 2002). Furthermore, the lack of uniform standards resulted in the absence of any guideline concerning the choice of neuropsychological instruments to be used to assess the patients' outcomes. It follows that, in some cases, the patients were presented with a very large battery of tests (e.g., Steinvorth et al., 2003; Welzel et al., 2008; Tsai et al., 2015; Brown et al., 2016) that encompass a very wide range of cognitive domains (i.e., memory, executive functions, language, abstract reasoning, etc.), while in other cases, authors focused on a specific domain and administered the patients with only a single test (e.g., Gondi et al., 2014: The Hopkins Verbal Learning Test-r; Welzel et al., 2008: The Auditory Verbal Learning Test), thus making a comparison between studies difficult, if not impossible. In particular, the absence of a widely acknowledged criterion to determine whether the patients' performance over the years should be considered worsened or improved so far prevented clinicians and epidemiologists to measure the exact prevalence of cognitive decline after cranial irradiation. Notwithstanding these limitations, there is a general consensus that memory is the cognitive function that is most often affected by cranial irradiation because of the involvement of the hippocampus. Actually, in the field of cognitive assessment, the individuals' performance on memory test is the one most influenced by practice. It follows that only pre-post clinical changes, where the effect of practice has been taken into account, should be judged as significant.

## Clinical Significance: the Reliable Change Index

One of the most common and critical issues within the literature about the cognitive consequences of cranial irradiation is the scarcity of attention to clinical significance and the statistical reliability of the measures employed. Indeed, many papers have assessed the pre–post changes only on the basis of the means of the pretreatment group and the posttreatment group. For example, neurocognitive dysfunction was alternatively defined as a ***z***-score drop of 1.5 (Gondi et al., 2012; Kim et al., 2018) or a decline of more than **two** standard deviations from the patient's own baseline value (Li et al., 2007). In other cases, the longitudinal trends of neurocognitive outcomes have been estimated by means of a regression analysis (Acharya et al., 2019).

However, these approaches are unable to assess if there is a clinical pre–post significant change for each individual. Following Zahra and Hedge (2010), an effect can be defined as clinically significant if “the individual has moved from being more like a clinical population […] to being more like a non-clinical population” (p. 15) or ***vice versa***. With regard to cranial irradiation, the point is to understand if an individual has moved to being more like a non-clinical population to be more similar to a clinical population in terms of neurocognitive deficits.

Another point to take into account is the reliability of the measure (i.e., its capability of producing similar results under consistent conditions). The commonly employed approach based on statistical significance usually does not take into account the reliability of the measures: however, many neuropsychological tests include highly reliable tests as well as not-so-reliable tests. In the latter case, the results should be interpreted in a more cautious manner.

The RCI developed by Jacobson and Truax (1991) [see also Christensen and Mendoza (1986) and Chelune et al. (1993)] is the gold-standard measure to assess statistical and clinical significance while taking into account the reliability of the neuropsychological test as well.

Assuming that the test–retest reliability coefficient *r*_xx_ and the standard deviation of the pretreatment group are known, it is possible to apply the RCI formula:

$$\begin{array}{l} \frac{t_{1}-t_{2}}{\sqrt{\left(2{\left(s_{1}\sqrt{1-r_{xx}}\right)}^{2}\right)}} \end{array}$$

where *t*_1_ and *t*_2_ are, respectively, the baseline and the posttreatment scores. Here Jacobson and Truax (1991) assume that two scores are parallel measures (the estimated common reliability coefficient and the common variance are obtained by the test–retest correlation and the variance of the initial measure in the current study; see Maassen, 2004). Computing the RCI is equivalent to computing a *z*-score; in other terms, it is a way to standardize the difference in SD units. According to basic statistics principles (Agresti and Finlay, 2009), an RC larger than 1.96 (in absolute value) would represent an actual change because it is unlikely to occur (*p* < 0.05) without an actual difference. In the field of neuropsychological assessment, a variant of the Jacobson and Truax (1991) formula proposed by Chelune et al. (1993) is more widespread: this alternative approach also takes into account practice effects as a consequence of repeated testing. There are other variants of the formula with different ways to calculate the standard error [for a discussion, see Hinton-Bayre (2000), Maassen (2004), and Temkin et al. (1999)]. According to several theoretical considerations of Maassen (2004), the preferable option is to assume that the reliability coefficients of pre- and post-measures are equal, estimating the former and the variances of the pre- and postscores from the research sample. Formally, the denominator can be computed as follows:

$$\begin{array}{l} \sqrt{\left(s_{1}^{2}+s_{2}^{2}\right)\left(1-r_{12}\right)} \end{array}$$

where the indexes 1 and 2 refer to the pre- and post-measures.

In the cranial irradiation literature, even if the RCI is not the standard approach to assess whether patients have been diagnosed as cognitively worsened (or improved), some studies have employed this index (Chang et al., 2009; Gondi et al., 2014; Tringale et al., 2019). For example, Gondi et al. (2014), on the basis of a version of RCI, adopted a drop of at least two, three, or five points from baseline on the Hopkins Verbal Learning Test—Revised (Benedict et al., 1998) for immediate recognition, delayed recall, and total recall, respectively.

The importance of the application of the RCI index within neuropsychology (including other ways of assessing clinical change) along with a discussion of common issues is reviewed in Duff (2012) [see also Temkin et al. (1999)]. Moreover, in some cases, the application of RCI has led to a controversy about the procedure outlined for determining confidence intervals (Abramson, 2000; Hinton-Bayre, 2000; Temkin et al., 2000; Maassen, 2004).

## Discussion

The aim of this contribution was to unsystematically highlight some methodological issues observed within the literature about the cognitive consequences of cranial irradiation for oncological management (Gondi et al., 2010, 2012, 2014; Kim et al., 2018; Acharya et al., 2019; Tringale et al., 2019). We advocate that much of the debate and many of the controversial results found within and across these studies are mainly based on such issues; the patients with disease of different clinical stages, the timing of neuropsychological assessment, and the absence of guidelines about the neuropsychological battery to employ for assessing the outcomes make it difficult to compare studies. In particular, we focus on the statistical criteria for determining cognitive deterioration (or improvement); the use of the RCI could be an ideal approach to assess a relevant clinical change in a pre–post comparison (Zahra and Hedge, 2010). However, not all the studies employed this approach, whereas many other works adopted traditional statistical analysis or arbitrary criteria.

The limited use of RCI does not concern only the literature about the cognitive deficit of cranial irradiation. Indeed, Zahra and Hedge (2010) asked themselves why RCI is not popular within academic psychology. According to them, possible explanations are the need for comparison of population means and standard deviations, the necessity to know the reliability of the measurements, and the fact that the most popular statistical packages do not compute it. With regard to the latter point, Zahra (2010) has made an RCI calculator available on his website.

It is important to note that we did not perform a systematic review of the literature: future review works should address the mentioned issues in order to produce a clear picture about the magnitude of such limitations and evaluate the impact about current conceptions on the cognitive effect of cranial irradiation.

To conclude, we believe that this work will induce greater awareness about the mentioned methodological issues and will contribute to a greater diffusion of RCI as a tool for determining clinical change in neuropsychological assessments performed as a result of cranial irradiation.

## Author Contributions

GG, SS, and AP equally contributed to all phases of the development of the manuscript including conception, literature review, and writing. All authors contributed to the article and approved the submitted version.

## Conflict of Interest

The authors declare that the research was conducted in the absence of any commercial or financial relationships that could be construed as a potential conflict of interest.
